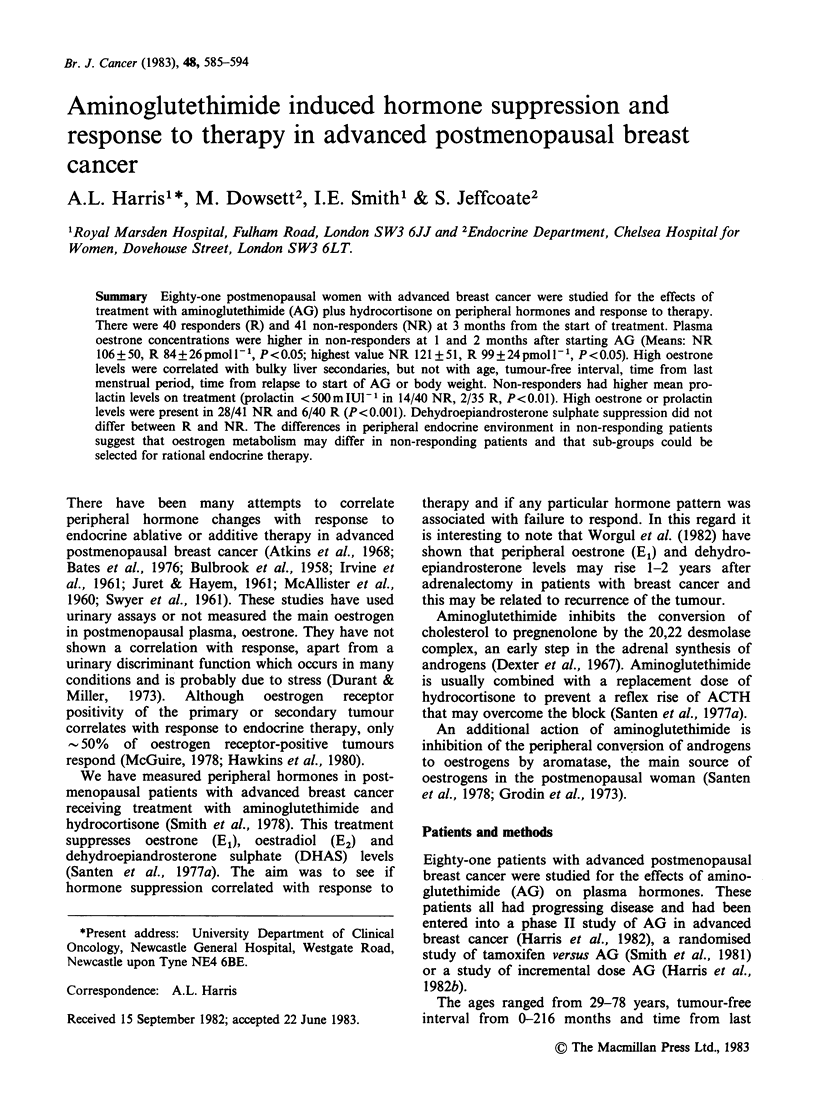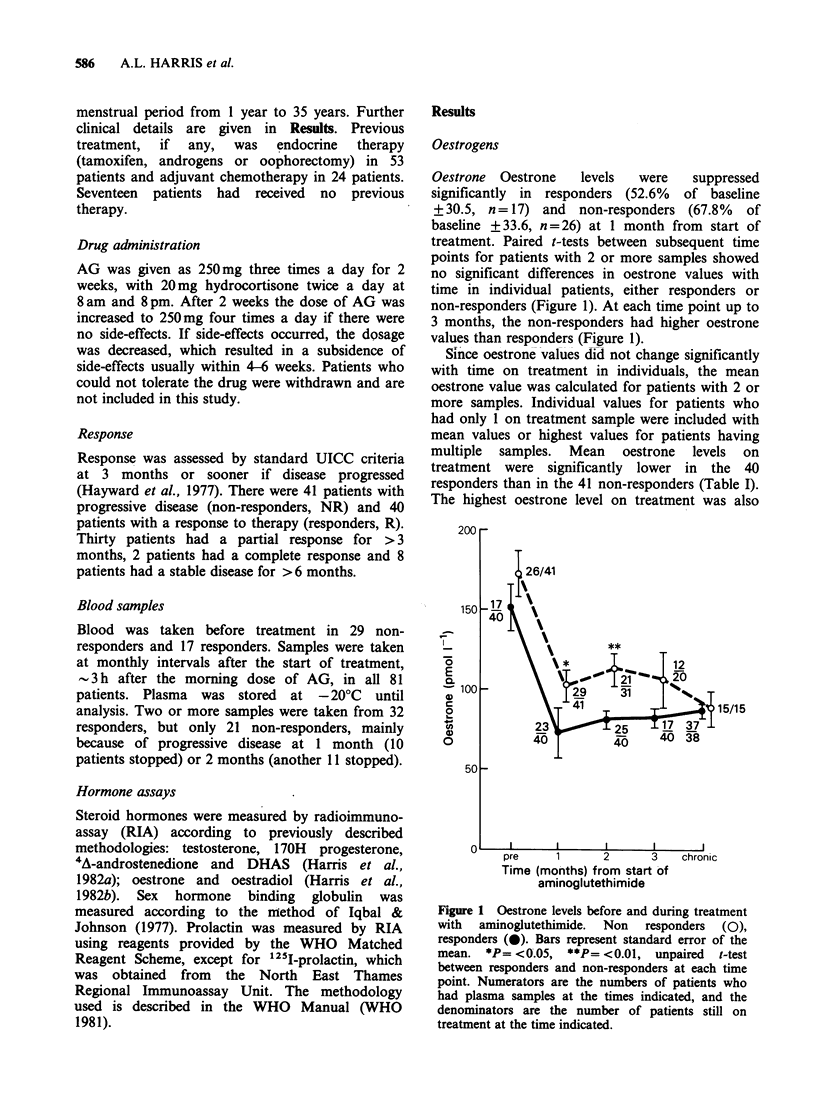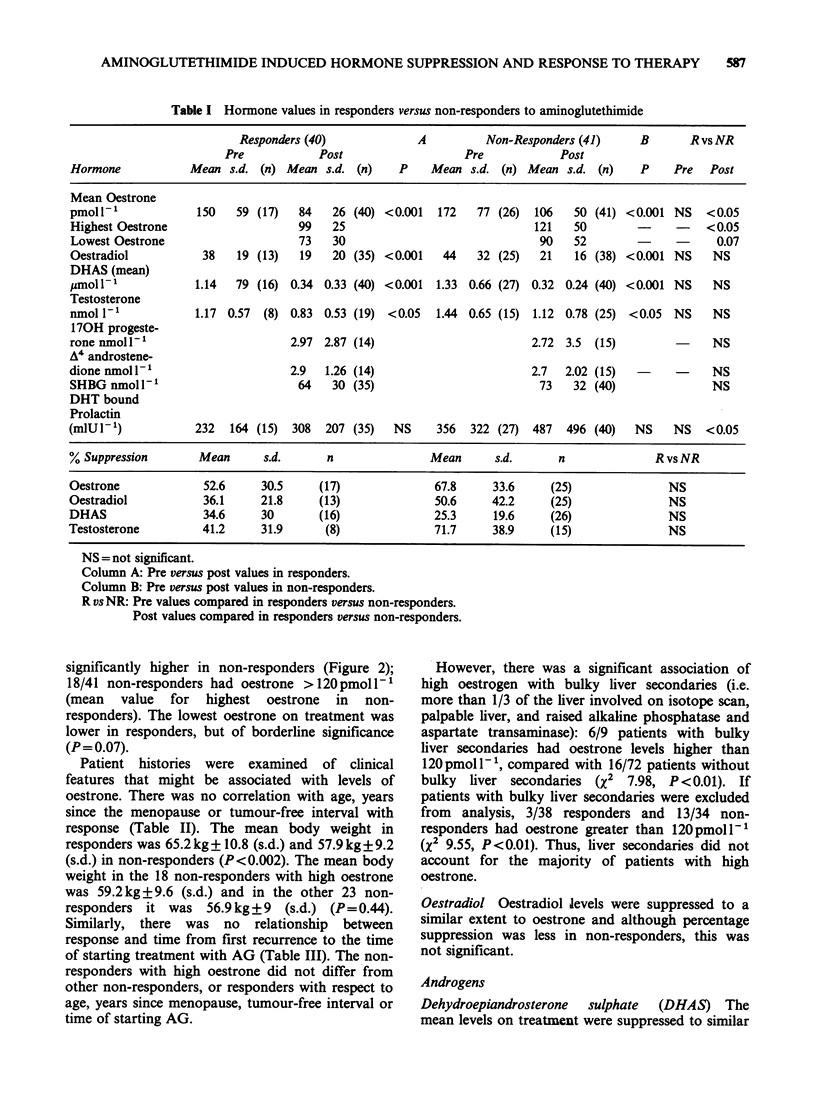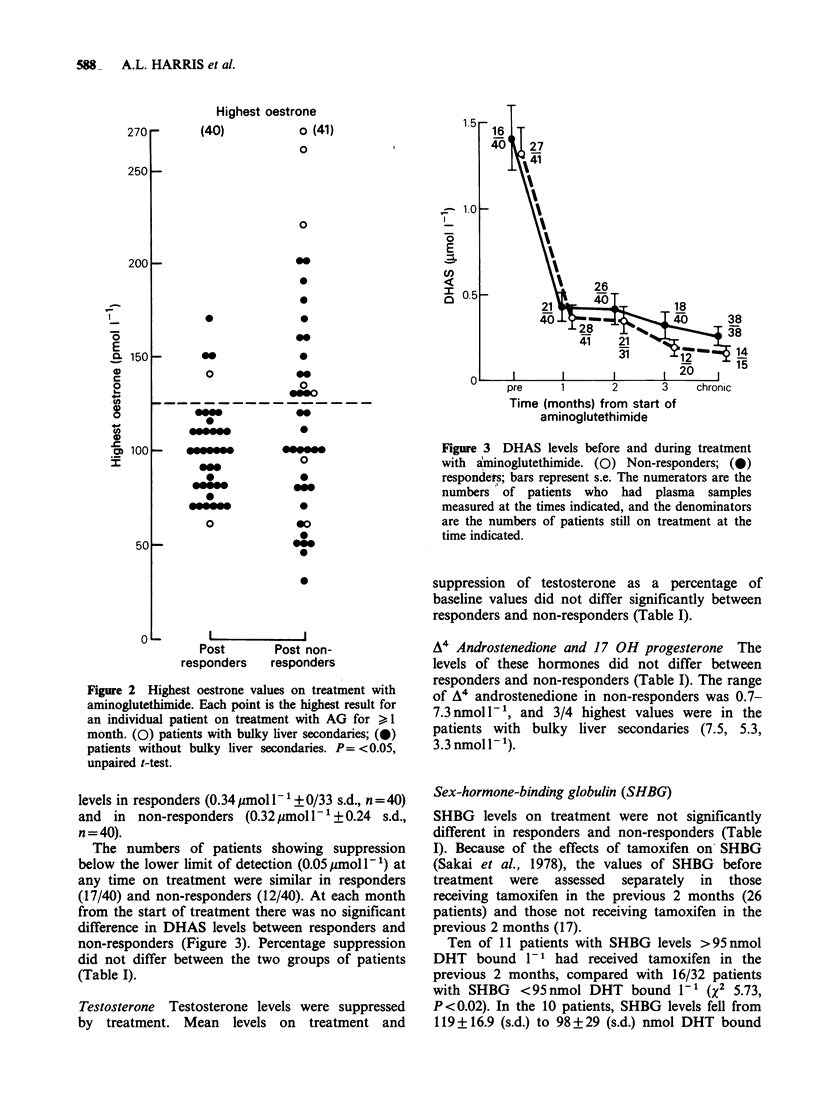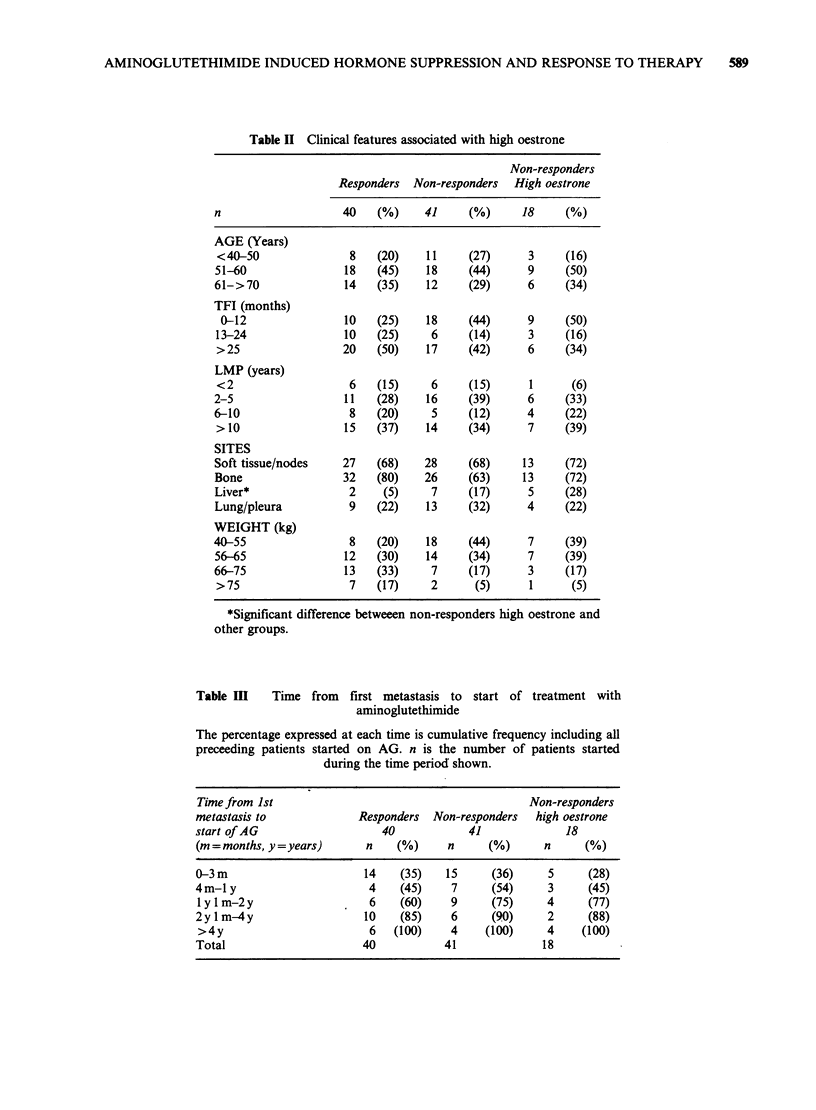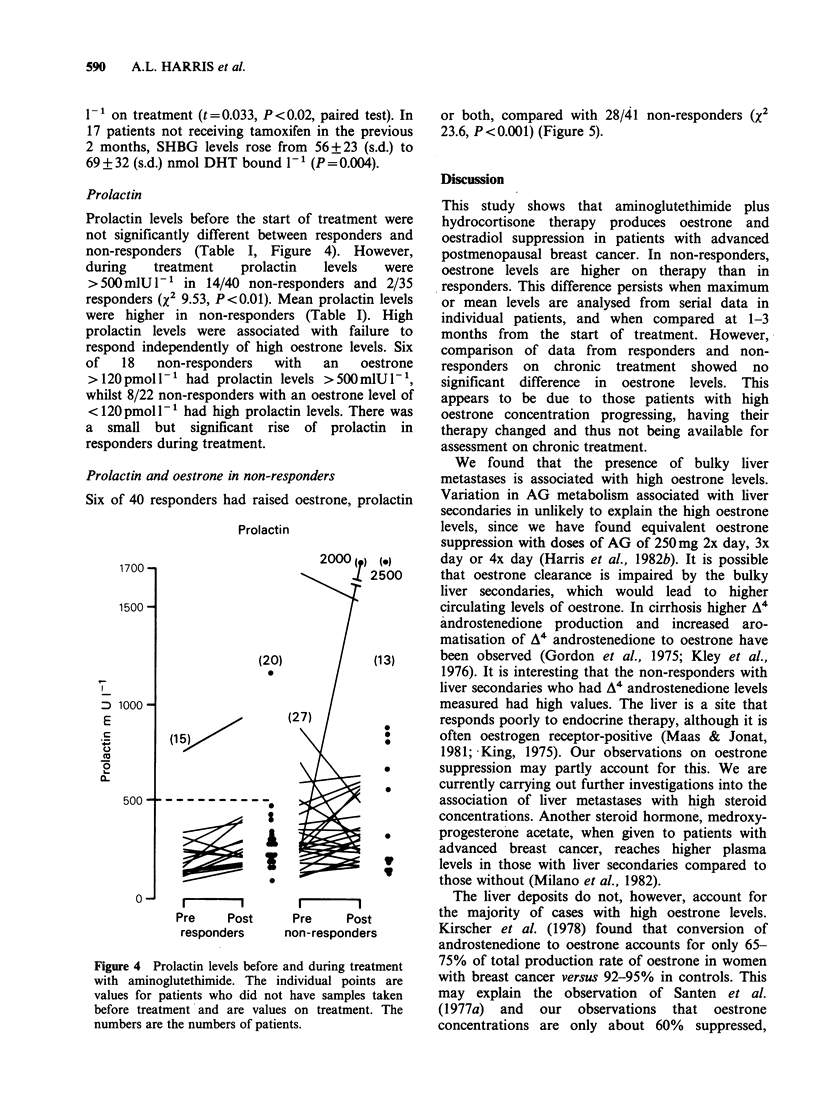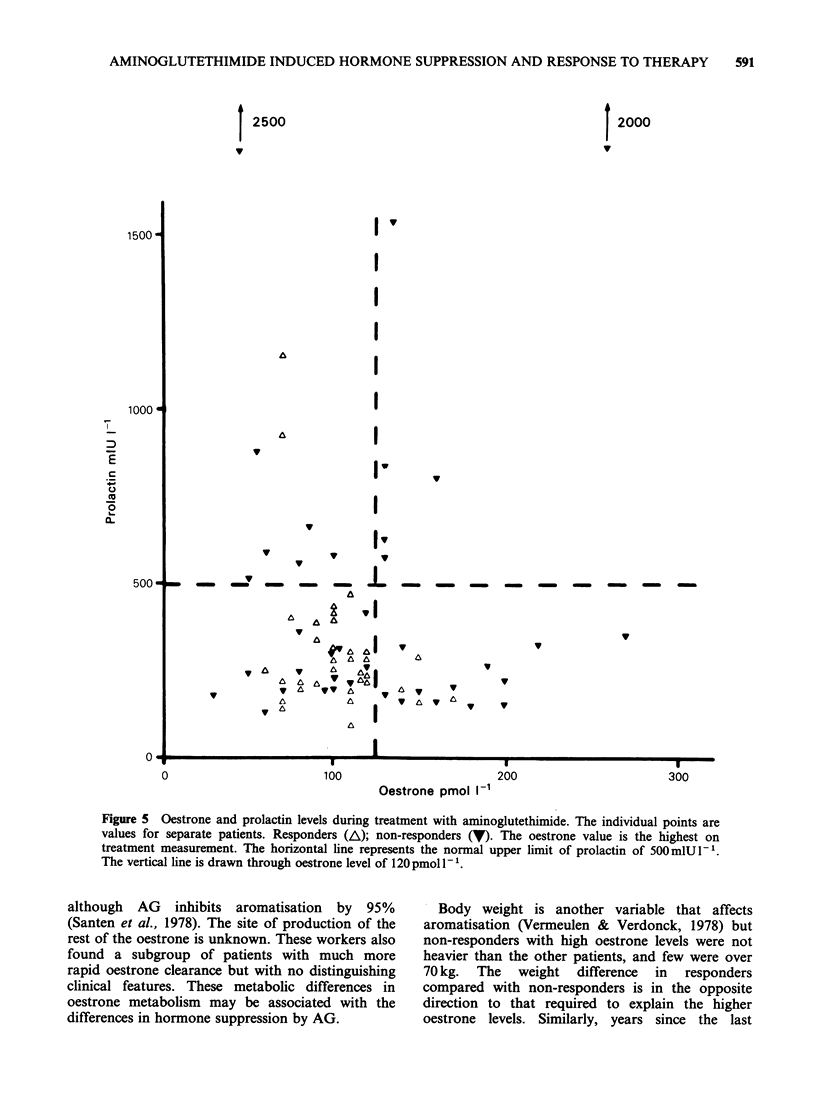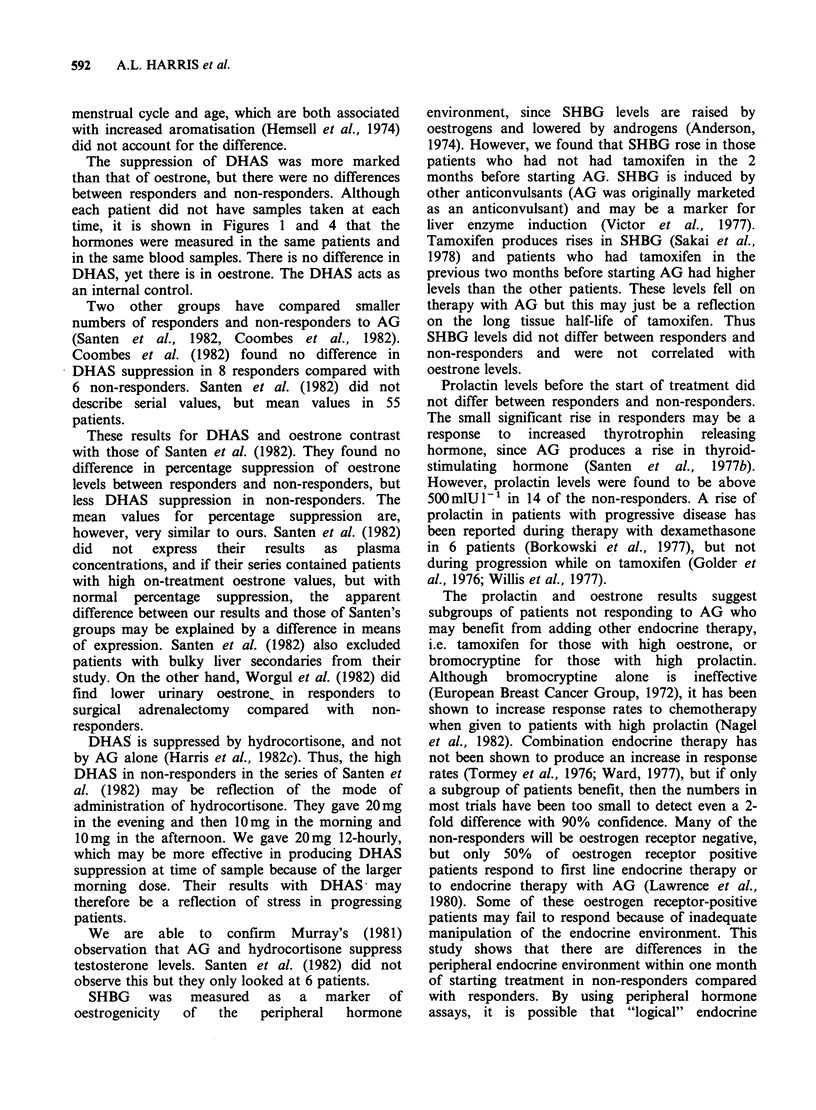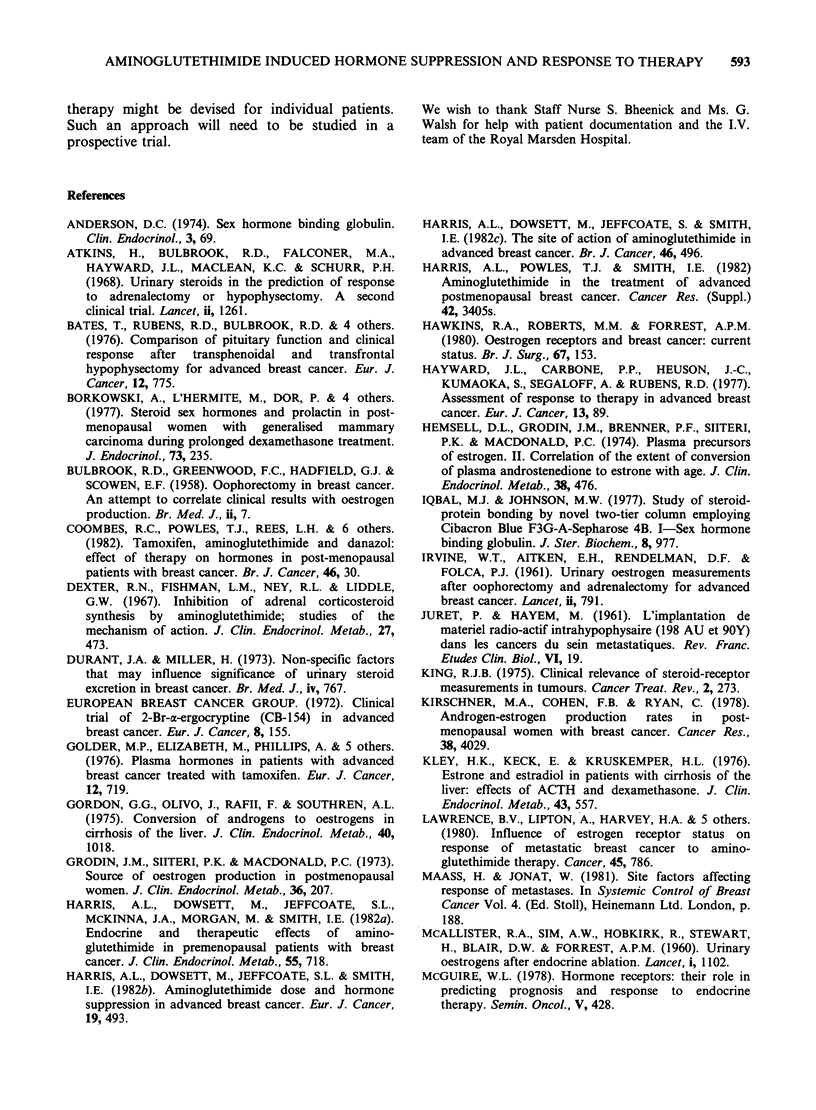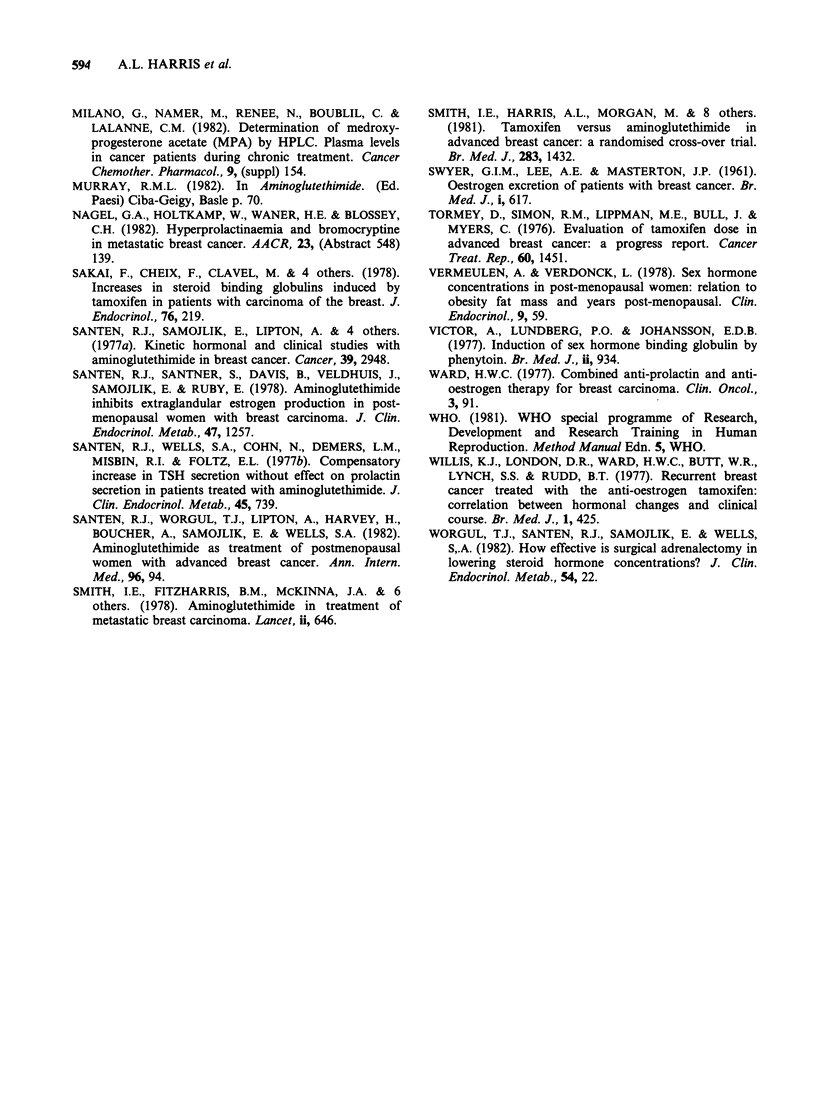# Aminoglutethimide induced hormone suppression and response to therapy in advanced postmenopausal breast cancer.

**DOI:** 10.1038/bjc.1983.232

**Published:** 1983-10

**Authors:** A. L. Harris, M. Dowsett, I. E. Smith, S. Jeffcoate

## Abstract

Eighty-one postmenopausal women with advanced breast cancer were studied for the effects of treatment with aminoglutethimide (AG) plus hydrocortisone on peripheral hormones and response to therapy. There were 40 responders (R) and 41 non-responders (NR) at 3 months from the start of treatment. Plasma oestrone concentrations were higher in non-responders at 1 and 2 months after starting AG (Means: NR 106 +/- 50, R 84 +/- 26 pmol l-1, P less than 0.05; highest value NR 121 +/- 51, R 99 +/- 24 pmol l-1, P less than 0.05). High oestrone levels were correlated with bulky liver secondaries, but not with age, tumour-free interval, time from last menstrual period, time from relapse to start of AG or body weight. Non-responders had higher mean prolactin levels on treatment (prolactin less than 500 mIUl-1 in 14/40 NR, 2/35 R, P less than 0.01). High oestrone or prolactin levels were present in 28/41 NR and 6/40 R (P less than 0.001). Dehydroepiandrosterone sulphate suppression did not differ between R and NR. The differences in peripheral endocrine environment in non-responding patients suggest that oestrogen metabolism may differ in non-responding patients and that sub-groups could be selected for rational endocrine therapy.


Br. J. Cancer (1983), 48, 585–594

### Aminoglutethimide induced hormone suppression and response to therapy in advanced postmenopausal breast cancer

A.L. Harris[1]*, M. Dowsett[2], I.E. Smith[1] & S. Jeffcoate[2]

[1]*Royal Marsden Hospital, Fulham Road, London SW3 6JJ and* [2]*Endocrine Department, Chelsea Hospital for Women, Dovehouse Street, London SW3 6LT.*

**Summary** Eighty-one postmenopausal women with advanced breast cancer were studied for the effects of treatment with aminoglutethimide (AG) plus hydrocortisone on peripheral hormones and response to therapy. There were 40 responders (R) and 41 non-responders (NR) at 3 months from the start of treatment. Plasma oestrone concentrations were higher in non-responders at 1 and 2 months after starting AG (Means: NR $106 \pm 50$, R $84 \pm 26\,pmol\,l^{-1}$, $P<0.05$; highest value NR $121 \pm 51$, R $99 \pm 24\,pmol\,l^{-1}$, $P<0.05$). High oestrone levels were correlated with bulky liver secondaries, but not with age, tumour-free interval, time from last menstrual period, time from relapse to start of AG or body weight. Non-responders had higher mean prolactin levels on treatment (prolactin $<500\,m\,IU\,l^{-1}$ in 14/40 NR, 2/35 R, $P<0.01$). High oestrone or prolactin levels were present in 28/41 NR and 6/40 R ($P<0.001$). Dehydroepiandrosterone sulphate suppression did not differ between R and NR. The differences in peripheral endocrine environment in non-responding patients suggest that oestrogen metabolism may differ in non-responding patients and that sub-groups could be selected for rational endocrine therapy.

There have been many attempts to correlate peripheral hormone changes with response to endocrine ablative or additive therapy in advanced postmenopausal breast cancer (Atkins *et al.*, 1968; Bates *et al.*, 1976; Bulbrook *et al.*, 1958; Irvine *et al.*, 1961; Juret & Hayem, 1961; McAllister *et al.*, 1960; Swyer *et al.*, 1961). These studies have used urinary assays or not measured the main oestrogen in postmenopausal plasma, oestrone. They have not shown a correlation with response, apart from a urinary discriminant function which occurs in many conditions and is probably due to stress (Durant & Miller, 1973). Although oestrogen receptor positivity of the primary or secondary tumour correlates with response to endocrine therapy, only ~50% of oestrogen receptor-positive tumours respond (McGuire, 1978; Hawkins *et al.*, 1980).

We have measured peripheral hormones in postmenopausal patients with advanced breast cancer receiving treatment with aminoglutethimide and hydrocortisone (Smith *et al.*, 1978). This treatment suppresses oestrone ($E_1$), oestradiol ($E_2$) and dehydroepiandrosterone sulphate (DHAS) levels (Santen *et al.*, 1977*a*). The aim was to see if hormone suppression correlated with response to therapy and if any particular hormone pattern was associated with failure to respond. In this regard it is interesting to note that Worgul *et al.* (1982) have shown that peripheral oestrone ($E_1$) and dehydroepiandrosterone levels may rise 1–2 years after adrenalectomy in patients with breast cancer and this may be related to recurrence of the tumour.

Aminoglutethimide inhibits the conversion of cholesterol to pregnenolone by the 20,22 desmolase complex, an early step in the adrenal synthesis of androgens (Dexter *et al.*, 1967). Aminoglutethimide is usually combined with a replacement dose of hydrocortisone to prevent a reflex rise of ACTH that may overcome the block (Santen *et al.*, 1977*a*).

An additional action of aminoglutethimide is inhibition of the peripheral conversion of androgens to oestrogens by aromatase, the main source of oestrogens in the postmenopausal woman (Santen *et al.*, 1978; Grodin *et al.*, 1973).

### Patients and methods

Eighty-one patients with advanced postmenopausal breast cancer were studied for the effects of aminoglutethimide (AG) on plasma hormones. These patients all had progressing disease and had been entered into a phase II study of AG in advanced breast cancer (Harris *et al.*, 1982), a randomised study of tamoxifen *versus* AG (Smith *et al.*, 1981) or a study of incremental dose AG (Harris *et al.*, 1982*b*).

The ages ranged from 29–78 years, tumour-free interval from 0–216 months and time from last

*Present address: University Department of Clinical Oncology, Newcastle General Hospital, Westgate Road, Newcastle upon Tyne NE4 6BE.

Correspondence: A.L. Harris

Received 15 September 1982; accepted 22 June 1983.

© The Macmillan Press Ltd., 1983

menstrual period from 1 year to 35 years. Further clinical details are given in **Results**. Previous treatment, if any, was endocrine therapy (tamoxifen, androgens or oophorectomy) in 53 patients and adjuvant chemotherapy in 24 patients. Seventeen patients had received no previous therapy.

*Drug administration*

AG was given as 250 mg three times a day for 2 weeks, with 20 mg hydrocortisone twice a day at 8 am and 8 pm. After 2 weeks the dose of AG was increased to 250 mg four times a day if there were no side-effects. If side-effects occurred, the dosage was decreased, which resulted in a subsidence of side-effects usually within 4–6 weeks. Patients who could not tolerate the drug were withdrawn and are not included in this study.

*Response*

Response was assessed by standard UICC criteria at 3 months or sooner if disease progressed (Hayward *et al.*, 1977). There were 41 patients with progressive disease (non-responders, NR) and 40 patients with a response to therapy (responders, R). Thirty patients had a partial response for >3 months, 2 patients had a complete response and 8 patients had a stable disease for >6 months.

*Blood samples*

Blood was taken before treatment in 29 non-responders and 17 responders. Samples were taken at monthly intervals after the start of treatment, ~3 h after the morning dose of AG, in all 81 patients. Plasma was stored at −20°C until analysis. Two or more samples were taken from 32 responders, but only 21 non-responders, mainly because of progressive disease at 1 month (10 patients stopped) or 2 months (another 11 stopped).

*Hormone assays*

Steroid hormones were measured by radioimmunoassay (RIA) according to previously described methodologies: testosterone, 17OH progesterone, $^4\Delta$-androstenedione and DHAS (Harris *et al.*, 1982*a*); oestrone and oestradiol (Harris *et al.*, 1982*b*). Sex hormone binding globulin was measured according to the method of Iqbal & Johnson (1977). Prolactin was measured by RIA using reagents provided by the WHO Matched Reagent Scheme, except for $^{125}$I-prolactin, which was obtained from the North East Thames Regional Immunoassay Unit. The methodology used is described in the WHO Manual (WHO 1981).

### Results

*Oestrogens*

*Oestrone* Oestrone levels were suppressed significantly in responders (52.6% of baseline ±30.5, $n=17$) and non-responders (67.8% of baseline ±33.6, $n=26$) at 1 month from start of treatment. Paired *t*-tests between subsequent time points for patients with 2 or more samples showed no significant differences in oestrone values with time in individual patients, either responders or non-responders (Figure 1). At each time point up to 3 months, the non-responders had higher oestrone values than responders (Figure 1).

Since oestrone values did not change significantly with time on treatment in individuals, the mean oestrone value was calculated for patients with 2 or more samples. Individual values for patients who had only 1 on treatment sample were included with mean values or highest values for patients having multiple samples. Mean oestrone levels on treatment were significantly lower in the 40 responders than in the 41 non-responders (Table I). The highest oestrone level on treatment was also

**Figure 1** Oestrone levels before and during treatment with aminoglutethimide. Non responders (○), responders (●). Bars represent standard error of the mean. $*P=<0.05$, $**P=<0.01$, unpaired *t*-test between responders and non-responders at each time point. Numerators are the numbers of patients who had plasma samples at the times indicated, and the denominators are the number of patients still on treatment at the time indicated.

**Table I** Hormone values in responders *versus* non-responders to aminoglutethimide

| | *Responders (40)* | | | | | | *A* | *Non-Responders (41)* | | | | | | *B* | *R* vs *NR* | |
|---|---|---|---|---|---|---|---|---|---|---|---|---|---|---|---|---|
| | *Pre* | | | *Post* | | | | *Pre* | | | *Post* | | | | | |
| *Hormone* | *Mean* | *s.d.* | *(n)* | *Mean* | *s.d.* | *(n)* | *P* | *Mean* | *s.d.* | *(n)* | *Mean* | *s.d.* | *(n)* | *P* | *Pre* | *Post* |
| Mean Oestrone pmol $l^{-1}$ | 150 | 59 | (17) | 84 | 26 | (40) | <0.001 | 172 | 77 | (26) | 106 | 50 | (41) | <0.001 | NS | <0.05 |
| Highest Oestrone | | | | 99 | 25 | | | | | | 121 | 50 | | — | — | <0.05 |
| Lowest Oestrone | | | | 73 | 30 | | | | | | 90 | 52 | | — | — | 0.07 |
| Oestradiol | 38 | 19 | (13) | 19 | 20 | (35) | <0.001 | 44 | 32 | (25) | 21 | 16 | (38) | <0.001 | NS | NS |
| DHAS (mean) $\mu$mol $l^{-1}$ | 1.14 | 79 | (16) | 0.34 | 0.33 | (40) | <0.001 | 1.33 | 0.66 | (27) | 0.32 | 0.24 | (40) | <0.001 | NS | NS |
| Testosterone nmol $l^{-1}$ | 1.17 | 0.57 | (8) | 0.83 | 0.53 | (19) | <0.05 | 1.44 | 0.65 | (15) | 1.12 | 0.78 | (25) | <0.05 | NS | NS |
| 17OH progesterone nmol $l^{-1}$ | | | | 2.97 | 2.87 | (14) | | | | | 2.72 | 3.5 | (15) | | — | NS |
| $\Delta^4$ androstenedione nmol $l^{-1}$ | | | | 2.9 | 1.26 | (14) | | | | | 2.7 | 2.02 | (15) | — | — | NS |
| SHBG nmol $l^{-1}$ DHT bound | | | | 64 | 30 | (35) | | | | | 73 | 32 | (40) | | | NS |
| Prolactin ($mIU\,l^{-1}$) | 232 | 164 | (15) | 308 | 207 | (35) | NS | 356 | 322 | (27) | 487 | 496 | (40) | NS | NS | <0.05 |

| *% Suppression* | *Mean* | *s.d.* | *n* | *Mean* | *s.d.* | *n* | *R* vs *NR* |
|---|---|---|---|---|---|---|---|
| Oestrone | 52.6 | 30.5 | (17) | 67.8 | 33.6 | (25) | NS |
| Oestradiol | 36.1 | 21.8 | (13) | 50.6 | 42.2 | (25) | NS |
| DHAS | 34.6 | 30 | (16) | 25.3 | 19.6 | (26) | NS |
| Testosterone | 41.2 | 31.9 | (8) | 71.7 | 38.9 | (15) | NS |

NS = not significant.
Column A: Pre *versus* post values in responders.
Column B: Pre *versus* post values in non-responders.
R *vs* NR: Pre values compared in responders *versus* non-responders.
Post values compared in responders *versus* non-responders.

significantly higher in non-responders (Figure 2); 18/41 non-responders had oestrone >120 pmol $l^{-1}$ (mean value for highest oestrone in non-responders). The lowest oestrone on treatment was lower in responders, but of borderline significance ($P$=0.07).

Patient histories were examined of clinical features that might be associated with levels of oestrone. There was no correlation with age, years since the menopause or tumour-free interval with response (Table II). The mean body weight in responders was 65.2 kg $\pm$ 10.8 (s.d.) and 57.9 kg $\pm$ 9.2 (s.d.) in non-responders ($P$<0.002). The mean body weight in the 18 non-responders with high oestrone was 59.2 kg $\pm$ 9.6 (s.d.) and in the other 23 non-responders it was 56.9 kg $\pm$ 9 (s.d.) ($P$=0.44). Similarly, there was no relationship between response and time from first recurrence to the time of starting treatment with AG (Table III). The non-responders with high oestrone did not differ from other non-responders, or responders with respect to age, years since menopause, tumour-free interval or time of starting AG.

However, there was a significant association of high oestrogen with bulky liver secondaries (i.e. more than 1/3 of the liver involved on isotope scan, palpable liver, and raised alkaline phosphatase and aspartate transaminase): 6/9 patients with bulky liver secondaries had oestrone levels higher than 120 pmol $l^{-1}$, compared with 16/72 patients without bulky liver secondaries ($\chi^2$ 7.98, $P$<0.01). If patients with bulky liver secondaries were excluded from analysis, 3/38 responders and 13/34 non-responders had oestrone greater than 120 pmol $l^{-1}$ ($\chi^2$ 9.55, $P$<0.01). Thus, liver secondaries did not account for the majority of patients with high oestrone.

*Oestradiol* Oestradiol levels were suppressed to a similar extent to oestrone and although percentage suppression was less in non-responders, this was not significant.

### *Androgens*

*Dehydroepiandrosterone sulphate* (*DHAS*) The mean levels on treatment were suppressed to similar

Figure 2 Highest oestrone values on treatment with aminoglutethimide. Each point is the highest result for an individual patient on treatment with AG for $\geqslant 1$ month. (○) patients with bulky liver secondaries; (●) patients without bulky liver secondaries. $P = <0.05$, unpaired $t$-test.

Figure 3 DHAS levels before and during treatment with aminoglutethimide. (○) Non-responders; (●) responders; bars represent s.e. The numerators are the numbers of patients who had plasma samples measured at the times indicated, and the denominators are the numbers of patients still on treatment at the time indicated.

levels in responders ($0.34\,\mu$mol l$^{-1}$ ±0/33 s.d., $n=40$) and in non-responders ($0.32\,\mu$mol l$^{-1}$ ±0.24 s.d., $n=40$).

The numbers of patients showing suppression below the lower limit of detection ($0.05\,\mu$mol l$^{-1}$) at any time on treatment were similar in responders (17/40) and non-responders (12/40). At each month from the start of treatment there was no significant difference in DHAS levels between responders and non-responders (Figure 3). Percentage suppression did not differ between the two groups of patients (Table I).

*Testosterone* Testosterone levels were suppressed by treatment. Mean levels on treatment and suppression of testosterone as a percentage of baseline values did not differ significantly between responders and non-responders (Table I).

*$\Delta^4$ Androstenedione and 17 OH progesterone* The levels of these hormones did not differ between responders and non-responders (Table I). The range of $\Delta^4$ androstenedione in non-responders was 0.7–7.3 nmol l$^{-1}$, and 3/4 highest values were in the patients with bulky liver secondaries (7.5, 5.3, 3.3 nmol l$^{-1}$).

*Sex-hormone-binding globulin (SHBG)*

SHBG levels on treatment were not significantly different in responders and non-responders (Table I). Because of the effects of tamoxifen on SHBG (Sakai *et al.*, 1978), the values of SHBG before treatment were assessed separately in those receiving tamoxifen in the previous 2 months (26 patients) and those not receiving tamoxifen in the previous 2 months (17).

Ten of 11 patients with SHBG levels >95 nmol DHT bound l$^{-1}$ had received tamoxifen in the previous 2 months, compared with 16/32 patients with SHBG <95 nmol DHT bound l$^{-1}$ ($\chi^2$ 5.73, $P<0.02$). In the 10 patients, SHBG levels fell from 119±16.9 (s.d.) to 98±29 (s.d.) nmol DHT bound

**Table II** Clinical features associated with high oestrone

| | *Responders* | | *Non-responders* | | *Non-responders High oestrone* | |
|---|---|---|---|---|---|---|
| *n* | 40 | (%) | *41* | (%) | *18* | (%) |
| AGE (Years) | | | | | | |
| <40–50 | 8 | (20) | 11 | (27) | 3 | (16) |
| 51–60 | 18 | (45) | 18 | (44) | 9 | (50) |
| 61–>70 | 14 | (35) | 12 | (29) | 6 | (34) |
| TFI (months) | | | | | | |
| 0–12 | 10 | (25) | 18 | (44) | 9 | (50) |
| 13–24 | 10 | (25) | 6 | (14) | 3 | (16) |
| >25 | 20 | (50) | 17 | (42) | 6 | (34) |
| LMP (years) | | | | | | |
| <2 | 6 | (15) | 6 | (15) | 1 | (6) |
| 2–5 | 11 | (28) | 16 | (39) | 6 | (33) |
| 6–10 | 8 | (20) | 5 | (12) | 4 | (22) |
| >10 | 15 | (37) | 14 | (34) | 7 | (39) |
| SITES | | | | | | |
| Soft tissue/nodes | 27 | (68) | 28 | (68) | 13 | (72) |
| Bone | 32 | (80) | 26 | (63) | 13 | (72) |
| Liver* | 2 | (5) | 7 | (17) | 5 | (28) |
| Lung/pleura | 9 | (22) | 13 | (32) | 4 | (22) |
| WEIGHT (kg) | | | | | | |
| 40–55 | 8 | (20) | 18 | (44) | 7 | (39) |
| 56–65 | 12 | (30) | 14 | (34) | 7 | (39) |
| 66–75 | 13 | (33) | 7 | (17) | 3 | (17) |
| >75 | 7 | (17) | 2 | (5) | 1 | (5) |

*Significant difference betweeen non-responders high oestrone and other groups.

**Table III** Time from first metastasis to start of treatment with aminoglutethimide

The percentage expressed at each time is cumulative frequency including all preceeding patients started on AG. *n* is the number of patients started during the time period shown.

| *Time from 1st metastasis to start of AG (m = months, y = years)* | *Responders 40* | | *Non-responders 41* | | *Non-responders high oestrone 18* | |
|---|---|---|---|---|---|---|
| | *n* | (%) | *n* | (%) | *n* | (%) |
| 0–3 m | 14 | (35) | 15 | (36) | 5 | (28) |
| 4 m–1 y | 4 | (45) | 7 | (54) | 3 | (45) |
| 1 y 1 m–2 y | 6 | (60) | 9 | (75) | 4 | (77) |
| 2 y 1 m–4 y | 10 | (85) | 6 | (90) | 2 | (88) |
| >4 y | 6 | (100) | 4 | (100) | 4 | (100) |
| Total | 40 | | 41 | | 18 | |

$l^{-1}$ on treatment ($t=0.033$, $P<0.02$, paired test). In 17 patients not receiving tamoxifen in the previous 2 months, SHBG levels rose from 56±23 (s.d.) to 69±32 (s.d.) nmol DHT bound $l^{-1}$ ($P=0.004$).

*Prolactin*

Prolactin levels before the start of treatment were not significantly different between responders and non-responders (Table I, Figure 4). However, during treatment prolactin levels were >500 mIU $l^{-1}$ in 14/40 non-responders and 2/35 responders ($\chi^2$ 9.53, $P<0.01$). Mean prolactin levels were higher in non-responders (Table I). High prolactin levels were associated with failure to respond independently of high oestrone levels. Six of 18 non-responders with an oestrone >120 pmol $l^{-1}$ had prolactin levels >500 mIU $l^{-1}$, whilst 8/22 non-responders with an oestrone level of <120 pmol $l^{-1}$ had high prolactin levels. There was a small but significant rise of prolactin in responders during treatment.

*Prolactin and oestrone in non-responders*

Six of 40 responders had raised oestrone, prolactin

**Figure 4** Prolactin levels before and during treatment with aminoglutethimide. The individual points are values for patients who did not have samples taken before treatment and are values on treatment. The numbers are the numbers of patients.

or both, compared with 28/41 non-responders ($\chi^2$ 23.6, $P<0.001$) (Figure 5).

### Discussion

This study shows that aminoglutethimide plus hydrocortisone therapy produces oestrone and oestradiol suppression in patients with advanced postmenopausal breast cancer. In non-responders, oestrone levels are higher on therapy than in responders. This difference persists when maximum or mean levels are analysed from serial data in individual patients, and when compared at 1–3 months from the start of treatment. However, comparison of data from responders and non-responders on chronic treatment showed no significant difference in oestrone levels. This appears to be due to those patients with high oestrone concentration progressing, having their therapy changed and thus not being available for assessment on chronic treatment.

We found that the presence of bulky liver metastases is associated with high oestrone levels. Variation in AG metabolism associated with liver secondaries in unlikely to explain the high oestrone levels, since we have found equivalent oestrone suppression with doses of AG of 250 mg 2x day, 3x day or 4x day (Harris *et al.*, 1982*b*). It is possible that oestrone clearance is impaired by the bulky liver secondaries, which would lead to higher circulating levels of oestrone. In cirrhosis higher $\Delta^4$ androstenedione production and increased aromatisation of $\Delta^4$ androstenedione to oestrone have been observed (Gordon *et al.*, 1975; Kley *et al.*, 1976). It is interesting that the non-responders with liver secondaries who had $\Delta^4$ androstenedione levels measured had high values. The liver is a site that responds poorly to endocrine therapy, although it is often oestrogen receptor-positive (Maas & Jonat, 1981; King, 1975). Our observations on oestrone suppression may partly account for this. We are currently carrying out further investigations into the association of liver metastases with high steroid concentrations. Another steroid hormone, medroxyprogesterone acetate, when given to patients with advanced breast cancer, reaches higher plasma levels in those with liver secondaries compared to those without (Milano *et al.*, 1982).

The liver deposits do not, however, account for the majority of cases with high oestrone levels. Kirscher *et al.* (1978) found that conversion of androstenedione to oestrone accounts for only 65–75% of total production rate of oestrone in women with breast cancer *versus* 92–95% in controls. This may explain the observation of Santen *et al.* (1977*a*) and our observations that oestrone concentrations are only about 60% suppressed,

**Figure 5** Oestrone and prolactin levels during treatment with aminoglutethimide. The individual points are values for separate patients. Responders (△); non-responders (▼). The oestrone value is the highest on treatment measurement. The horizontal line represents the normal upper limit of prolactin of 500 mIU l$^{-1}$. The vertical line is drawn through oestrone level of 120 pmol l$^{-1}$.

although AG inhibits aromatisation by 95% (Santen *et al.*, 1978). The site of production of the rest of the oestrone is unknown. These workers also found a subgroup of patients with much more rapid oestrone clearance but with no distinguishing clinical features. These metabolic differences in oestrone metabolism may be associated with the differences in hormone suppression by AG.

Body weight is another variable that affects aromatisation (Vermeulen & Verdonck, 1978) but non-responders with high oestrone levels were not heavier than the other patients, and few were over 70 kg. The weight difference in responders compared with non-responders is in the opposite direction to that required to explain the higher oestrone levels. Similarly, years since the last menstrual cycle and age, which are both associated with increased aromatisation (Hemsell *et al.*, 1974) did not account for the difference.

The suppression of DHAS was more marked than that of oestrone, but there were no differences between responders and non-responders. Although each patient did not have samples taken at each time, it is shown in Figures 1 and 4 that the hormones were measured in the same patients and in the same blood samples. There is no difference in DHAS, yet there is in oestrone. The DHAS acts as an internal control.

Two other groups have compared smaller numbers of responders and non-responders to AG (Santen *et al.*, 1982, Coombes *et al.*, 1982). Coombes *et al.* (1982) found no difference in DHAS suppression in 8 responders compared with 6 non-responders. Santen *et al.* (1982) did not describe serial values, but mean values in 55 patients.

These results for DHAS and oestrone contrast with those of Santen *et al.* (1982). They found no difference in percentage suppression of oestrone levels between responders and non-responders, but less DHAS suppression in non-responders. The mean values for percentage suppression are, however, very similar to ours. Santen *et al.* (1982) did not express their results as plasma concentrations, and if their series contained patients with high on-treatment oestrone values, but with normal percentage suppression, the apparent difference between our results and those of Santen's groups may be explained by a difference in means of expression. Santen *et al.* (1982) also excluded patients with bulky liver secondaries from their study. On the other hand, Worgul *et al.* (1982) did find lower urinary oestrone in responders to surgical adrenalectomy compared with non-responders.

DHAS is suppressed by hydrocortisone, and not by AG alone (Harris *et al.*, 1982*c*). Thus, the high DHAS in non-responders in the series of Santen *et al.* (1982) may be reflection of the mode of administration of hydrocortisone. They gave 20 mg in the evening and then 10 mg in the morning and 10 mg in the afternoon. We gave 20 mg 12-hourly, which may be more effective in producing DHAS suppression at time of sample because of the larger morning dose. Their results with DHAS may therefore be a reflection of stress in progressing patients.

We are able to confirm Murray's (1981) observation that AG and hydrocortisone suppress testosterone levels. Santen *et al.* (1982) did not observe this but they only looked at 6 patients.

SHBG was measured as a marker of oestrogenicity of the peripheral hormone environment, since SHBG levels are raised by oestrogens and lowered by androgens (Anderson, 1974). However, we found that SHBG rose in those patients who had not had tamoxifen in the 2 months before starting AG. SHBG is induced by other anticonvulsants (AG was originally marketed as an anticonvulsant) and may be a marker for liver enzyme induction (Victor *et al.*, 1977). Tamoxifen produces rises in SHBG (Sakai *et al.*, 1978) and patients who had tamoxifen in the previous two months before starting AG had higher levels than the other patients. These levels fell on therapy with AG but this may just be a reflection on the long tissue half-life of tamoxifen. Thus SHBG levels did not differ between responders and non-responders and were not correlated with oestrone levels.

Prolactin levels before the start of treatment did not differ between responders and non-responders. The small significant rise in responders may be a response to increased thyrotrophin releasing hormone, since AG produces a rise in thyroid-stimulating hormone (Santen *et al.*, 1977*b*). However, prolactin levels were found to be above 500 mIU $l^{-1}$ in 14 of the non-responders. A rise of prolactin in patients with progressive disease has been reported during therapy with dexamethasone in 6 patients (Borkowski *et al.*, 1977), but not during progression while on tamoxifen (Golder *et al.*, 1976; Willis *et al.*, 1977).

The prolactin and oestrone results suggest subgroups of patients not responding to AG who may benefit from adding other endocrine therapy, i.e. tamoxifen for those with high oestrone, or bromocryptine for those with high prolactin. Although bromocryptine alone is ineffective (European Breast Cancer Group, 1972), it has been shown to increase response rates to chemotherapy when given to patients with high prolactin (Nagel *et al.*, 1982). Combination endocrine therapy has not been shown to produce an increase in response rates (Tormey *et al.*, 1976; Ward, 1977), but if only a subgroup of patients benefit, then the numbers in most trials have been too small to detect even a 2-fold difference with 90% confidence. Many of the non-responders will be oestrogen receptor negative, but only 50% of oestrogen receptor positive patients respond to first line endocrine therapy or to endocrine therapy with AG (Lawrence *et al.*, 1980). Some of these oestrogen receptor-positive patients may fail to respond because of inadequate manipulation of the endocrine environment. This study shows that there are differences in the peripheral endocrine environment within one month of starting treatment in non-responders compared with responders. By using peripheral hormone assays, it is possible that "logical" endocrine therapy might be devised for individual patients. Such an approach will need to be studied in a prospective trial.

We wish to thank Staff Nurse S. Bheenick and Ms. G. Walsh for help with patient documentation and the I.V. team of the Royal Marsden Hospital.

### References

ANDERSON, D.C. (1974). Sex hormone binding globulin. *Clin. Endocrinol.*, **3**, 69.

ATKINS, H., BULBROOK, R.D., FALCONER, M.A., HAYWARD, J.L., MACLEAN, K.C. & SCHURR, P.H. (1968). Urinary steroids in the prediction of response to adrenalectomy or hypophysectomy. A second clinical trial. *Lancet*, **ii**, 1261.

BATES, T., RUBENS, R.D., BULBROOK, R.D. & 4 others. (1976). Comparison of pituitary function and clinical response after transphenoidal and transfrontal hypophysectomy for advanced breast cancer. *Eur. J. Cancer*, **12**, 775.

BORKOWSKI, A., L'HERMITE, M., DOR, P. & 4 others. (1977). Steroid sex hormones and prolactin in post-menopausal women with generalised mammary carcinoma during prolonged dexamethasone treatment. *J. Endocrinol.*, **73**, 235.

BULBROOK, R.D., GREENWOOD, F.C., HADFIELD, G.J. & SCOWEN, E.F. (1958). Oophorectomy in breast cancer. An attempt to correlate clinical results with oestrogen production. *Br. Med. J.*, **ii**, 7.

COOMBES, R.C., POWLES, T.J., REES, L.H. & 6 others. (1982). Tamoxifen, aminoglutethimide and danazol: effect of therapy on hormones in post-menopausal patients with breast cancer. *Br. J. Cancer*, **46**, 30.

DEXTER, R.N., FISHMAN, L.M., NEY, R.L. & LIDDLE, G.W. (1967). Inhibition of adrenal corticosteroid synthesis by aminoglutethimide; studies of the mechanism of action. *J. Clin. Endocrinol. Metab.*, **27**, 473.

DURANT, J.A. & MILLER, H. (1973). Non-specific factors that may influence significance of urinary steroid excretion in breast cancer. *Br. Med. J.*, **iv**, 767.

EUROPEAN BREAST CANCER GROUP. (1972). Clinical trial of 2-Br-α-ergocryptine (CB-154) in advanced breast cancer. *Eur. J. Cancer*, **8**, 155.

GOLDER, M.P., ELIZABETH, M., PHILLIPS, A. & 5 others. (1976). Plasma hormones in patients with advanced breast cancer treated with tamoxifen. *Eur. J. Cancer*, **12**, 719.

GORDON, G.G., OLIVO, J., RAFII, F. & SOUTHREN, A.L. (1975). Conversion of androgens to oestrogens in cirrhosis of the liver. *J. Clin. Endocrinol. Metab.*, **40**, 1018.

GRODIN, J.M., SIITERI, P.K. & MACDONALD, P.C. (1973). Source of oestrogen production in postmenopausal women. *J. Clin. Endocrinol. Metab.*, **36**, 207.

HARRIS, A.L., DOWSETT, M., JEFFCOATE, S.L., MCKINNA, J.A., MORGAN, M. & SMITH, I.E. (1982*a*). Endocrine and therapeutic effects of amino-glutethimide in premenopausal patients with breast cancer. *J. Clin. Endocrinol. Metab.*, **55**, 718.

HARRIS, A.L., DOWSETT, M., JEFFCOATE, S.L. & SMITH, I.E. (1982*b*). Aminoglutethimide dose and hormone suppression in advanced breast cancer. *Eur. J. Cancer*, **19**, 493.

HARRIS, A.L., DOWSETT, M., JEFFCOATE, S. & SMITH, I.E. (1982*c*). The site of action of aminoglutethimide in advanced breast cancer. *Br. J. Cancer*, **46**, 496.

HARRIS, A.L., POWLES, T.J. & SMITH, I.E. (1982) Aminoglutethimide in the treatment of advanced postmenopausal breast cancer. *Cancer Res.* (Suppl.) **42**, 3405s.

HAWKINS, R.A., ROBERTS, M.M. & FORREST, A.P.M. (1980). Oestrogen receptors and breast cancer: current status. *Br. J. Surg.*, **67**, 153.

HAYWARD, J.L., CARBONE, P.P., HEUSON, J.-C., KUMAOKA, S., SEGALOFF, A. & RUBENS, R.D. (1977). Assessment of response to therapy in advanced breast cancer. *Eur. J. Cancer*, **13**, 89.

HEMSELL, D.L., GRODIN, J.M., BRENNER, P.F., SIITERI, P.K. & MACDONALD, P.C. (1974). Plasma precursors of estrogen. II. Correlation of the extent of conversion of plasma androstenedione to estrone with age. *J. Clin. Endocrinol. Metab.*, **38**, 476.

IQBAL, M.J. & JOHNSON, M.W. (1977). Study of steroid-protein bonding by novel two-tier column employing Cibacron Blue F3G-A-Sepharose 4B. I—Sex hormone binding globulin. *J. Ster. Biochem.*, **8**, 977.

IRVINE, W.T., AITKEN, E.H., RENDELMAN, D.F. & FOLCA, P.J. (1961). Urinary oestrogen measurements after oophorectomy and adrenalectomy for advanced breast cancer. *Lancet*, **ii**, 791.

JURET, P. & HAYEM, M. (1961). L'implantation de materiel radio-actif intrahypophysaire (198 AU et 90Y) dans les cancers du sein metastatiques. *Rev. Franc. Etudes Clin. Biol.*, **VI**, 19.

KING, R.J.B. (1975). Clinical relevance of steroid-receptor measurements in tumours. *Cancer Treat. Rev.*, **2**, 273.

KIRSCHNER, M.A., COHEN, F.B. & RYAN, C. (1978). Androgen-estrogen production rates in post-menopausal women with breast cancer. *Cancer Res.*, **38**, 4029.

KLEY, H.K., KECK, E. & KRUSKEMPER, H.L. (1976). Estrone and estradiol in patients with cirrhosis of the liver: effects of ACTH and dexamethasone. *J. Clin. Endocrinol. Metab.*, **43**, 557.

LAWRENCE, B.V., LIPTON, A., HARVEY, H.A. & 5 others. (1980). Influence of estrogen receptor status on response of metastatic breast cancer to amino-glutethimide therapy. *Cancer*, **45**, 786.

MAASS, H. & JONAT, W. (1981). Site factors affecting response of metastases. In *Systemic Control of Breast Cancer* Vol. 4. (Ed. Stoll), Heinemann Ltd. London, p. 188.

MCALLISTER, R.A., SIM, A.W., HOBKIRK, R., STEWART, H., BLAIR, D.W. & FORREST, A.P.M. (1960). Urinary oestrogens after endocrine ablation. *Lancet*, **i**, 1102.

MCGUIRE, W.L. (1978). Hormone receptors: their role in predicting prognosis and response to endocrine therapy. *Semin. Oncol.*, **V**, 428.

MILANO, G., NAMER, M., RENEE, N., BOUBLIL, C. & LALANNE, C.M. (1982). Determination of medroxyprogesterone acetate (MPA) by HPLC. Plasma levels in cancer patients during chronic treatment. *Cancer Chemother. Pharmacol.*, **9**, (suppl) 154.

MURRAY, R.M.L. (1982). In *Aminoglutethimide*. (Ed. Paesi) Ciba-Geigy, Basle p. 70.

NAGEL, G.A., HOLTKAMP, W., WANER, H.E. & BLOSSEY, C.H. (1982). Hyperprolactinaemia and bromocryptine in metastatic breast cancer. *AACR*, **23**, (Abstract 548) 139.

SAKAI, F., CHEIX, F., CLAVEL, M. & 4 others. (1978). Increases in steroid binding globulins induced by tamoxifen in patients with carcinoma of the breast. *J. Endocrinol.*, **76**, 219.

SANTEN, R.J., SAMOJLIK, E., LIPTON, A. & 4 others. (1977*a*). Kinetic hormonal and clinical studies with aminoglutethimide in breast cancer. *Cancer*, **39**, 2948.

SANTEN, R.J., SANTNER, S., DAVIS, B., VELDHUIS, J., SAMOJLIK, E. & RUBY, E. (1978). Aminoglutethimide inhibits extraglandular estrogen production in postmenopausal women with breast carcinoma. *J. Clin. Endocrinol. Metab.*, **47**, 1257.

SANTEN, R.J., WELLS, S.A., COHN, N., DEMERS, L.M., MISBIN, R.I. & FOLTZ, E.L. (1977*b*). Compensatory increase in TSH secretion without effect on prolactin secretion in patients treated with aminoglutethimide. *J. Clin. Endocrinol. Metab.*, **45**, 739.

SANTEN, R.J., WORGUL, T.J., LIPTON, A., HARVEY, H., BOUCHER, A., SAMOJLIK, E. & WELLS, S.A. (1982). Aminoglutethimide as treatment of postmenopausal women with advanced breast cancer. *Ann. Intern. Med.*, **96**, 94.

SMITH, I.E., FITZHARRIS, B.M., McKINNA, J.A. & 6 others. (1978). Aminoglutethimide in treatment of metastatic breast carcinoma. *Lancet*, **ii**, 646.

SMITH, I.E., HARRIS, A.L., MORGAN, M. & 8 others. (1981). Tamoxifen versus aminoglutethimide in advanced breast cancer: a randomised cross-over trial. *Br. Med. J.*, **283**, 1432.

SWYER, G.I.M., LEE, A.E. & MASTERTON, J.P. (1961). Oestrogen excretion of patients with breast cancer. *Br. Med. J.*, **i**, 617.

TORMEY, D., SIMON, R.M., LIPPMAN, M.E., BULL, J. & MYERS, C. (1976). Evaluation of tamoxifen dose in advanced breast cancer: a progress report. *Cancer Treat. Rep.*, **60**, 1451.

VERMEULEN, A. & VERDONCK, L. (1978). Sex hormone concentrations in post-menopausal women: relation to obesity fat mass and years post-menopausal. *Clin. Endocrinol.*, **9**, 59.

VICTOR, A., LUNDBERG, P.O. & JOHANSSON, E.D.B. (1977). Induction of sex hormone binding globulin by phenytoin. *Br. Med. J.*, **ii**, 934.

WARD, H.W.C. (1977). Combined anti-prolactin and anti-oestrogen therapy for breast carcinoma. *Clin. Oncol.*, **3**, 91.

WHO. (1981). WHO special programme of Research, Development and Research Training in Human Reproduction. *Method Manual* Edn. **5**, WHO.

WILLIS, K.J., LONDON, D.R., WARD, H.W.C., BUTT, W.R., LYNCH, S.S. & RUDD, B.T. (1977). Recurrent breast cancer treated with the anti-oestrogen tamoxifen: correlation between hormonal changes and clinical course. *Br. Med. J.*, **1**, 425.

WORGUL, T.J., SANTEN, R.J., SAMOJLIK, E. & WELLS, S,.A. (1982). How effective is surgical adrenalectomy in lowering steroid hormone concentrations? *J. Clin. Endocrinol. Metab.*, **54**, 22.